# Older age is a risk factor associated with poor prognosis of patients with squamous cell carcinoma of the oral cavity

**DOI:** 10.1007/s00405-020-05963-3

**Published:** 2020-04-22

**Authors:** Shuwei Chen, Zhu Lin, Jingtao Chen, Ankui Yang, Quan Zhang, Chuanbo Xie, Xing Zhang, Zhongyuan Yang, Wenkuan Chen, Ming Song

**Affiliations:** 1 Department of Head and Neck Surgery, Sun Yat-Sen University Cancer Center, 651 Dongfeng Dong Road, Guangzhou, 510060 Guangdong China; 2 State Key Laboratory of Oncology in South China, Sun Yat-Sen University Cancer Center, 651 Dongfeng Dong Road, Guangzhou, 510060 Guangdong China; 3 Collaborative Innovation Center for Cancer Medicine, Sun Yat-Sen University Cancer Center, 651 Dongfeng Dong Road, Guangzhou, 510060 Guangdong China; 4 Cancer Prevention Center, Sun Yat-Sen University Cancer Center, 651 Dongfeng Dong Road, Guangzhou, 510060 Guangdong China

**Keywords:** Young, Squamous cell carcinoma, Oral cavity, Survival

## Abstract

**Purpose:**

Whether or not young patients with squamous cell carcinoma of oral cavity (OC-SCC) have a difference in prognosis remains a controversy. This study aimed to analyze the clinical characteristics and difference of survival rates between adult patients less than 40 years of age and those 40 years of age and older.

**Methods:**

A retrospective analysis was conducted using the database of patients diagnosed with OC-SCC between 1990 and 2013 in the Sun Yat-sen University Cancer Center, but patients older than 85 years, younger than 18 years, or died within 6 months of diagnosis were excluded. Patients were categorized into two groups: the young group (< 40 years of age) and the older group (≥ 40 years of age). Cox regression, survival and subgroups analyses were performed. The primary endpoints included the rates of 5-year overall survival (OS) and disease-specific survival (DSS).

**Results:**

A total of 1902 OC-SCC patients were identified. The percentage of female in the young group was significantly higher than that in the older group (40.27% vs 31.03%, *p* < 0.001). This study failed to find the difference in TNM classification or tumor stage between the two groups (*p* > 0.05). The young group was more likely to receive adjuvant radiotherapy and/or chemotherapy (42.48% vs 26.91%, *p* < 0.001). The 5-year OS rate (71% vs. 57%, *p* < 0.001) and DSS rate (72% vs 58%, *p* < 0.001) in patients under 40 years were significantly higher than those for the older group.

**Conclusion:**

Our findings suggested that OC-SCC in younger patients did not present at a more advanced stage. In addition, young age is an independent predictor for better survival.

## Introduction

Oral cavity cancer (OCC), which anatomically involves the lips, the front two-thirds of the tongue, the gums, the lining inside the cheeks and lips, the floor (bottom) of the mouth under the tongue, the hard palate (bony top of the mouth), and the small area of the gum behind the wisdom teeth [1], is one of the most common subsites of head and neck cancer [2]. Over 90% of cases are squamous cell carcinomas (SCC) [2]. Alcohol and tobacco consumption are considered to be the main risk factors for OCC [4, 5]. Further, OCC was found to be closely related to a high-risk human papillomavirus (HPV) infection [6]. In many parts of Asia, betel quid chewing increases the risk of oral cancer, independently of tobacco and alcohol use [7]. Worldwide, it is estimated that there will be 354,864 new cases of oral cancer and an estimated 177,384 people will die of the disease in 2018, representing close to 2% of cancer deaths. The incidence and mortality rates in men are approximately two times higher than those in women. Notably, OCC tends to cluster in South Asia [7]. In the United States, the incidence rate is highest in individuals aged 55–64 years, with a median age of 63 years. Data estimates from the US for the years 2009–2015 showed that the number of surviving patients of oral cavity and pharynx cancer at 5 years is 65.3%. Most patients (93.4%) are diagnosed at age 45 and above [9]. Traditionally, OCC occurs in the elderly during the 5th through the 7th decades of life; however, it has been reported to be increasing in incidence among younger populations globally, especially in young women [11].

The cause of this increasing trend remains unclear, but we should draw attention to the younger patients. Many published reports have obtained conflicting results. Some studies report that OC-SCC in young patients is more aggressive. Conversely, other studies suggest that younger patients with OC-SCC did not have worse survival. Whether or not the young with OC-SCC have a difference in prognosis remains a controversy. In this single-institution study performed in southern China, we aimed to compare differences in survival between patients aged younger than 40 years and those who were 40 years and older.

## Materials and methods

The study was conducted in Sun Yat-sen University Cancer Center (SYSUCC). The medical files, containing a total of 1902 patients who were treated primarily by surgical resection and histologically confirmed in our center from 1990 to 2013, were retrospectively reviewed. All the patients were followed for a minimum of 5 years. Patients were divided into two groups depending on age (patients < 40 years and patients ≥ 40 years). Demographics (age and sex), year of diagnosis (1990–1999, 2000–2009, 2010–2013), habits (alcohol and tobacco use), TNM classification, tumor stage (followed the AJCC Cancer Staging Manual, Seventh Edition), treatment (surgery, chemotherapy, radiotherapy), and survival outcomes were documented (see Table 1), and these factors were compared between patients younger than 40 years and the older patients. *P* < 0.01 was considered to be statistically significant. For statistical analyses, we used SPSS, version 16.0 software (SPSS Inc., Chicago, IL, USA).

**Table 1:** Patient characteristics

	All patients		Young group (< 40)		Older group (40–85)		
Characteristics	Number	%	Number	%	Number	%	*p* value
Total	1902	100	226	100	1676	100	
Age: median ± SD	54.6 ± 12.2		33.5 ± 4.7		57.5 ± 9.9		
Sex							< 0.001
Male	1291	67.88	135	59.73	1156	68.97	
Female	611	32.12	91	40.27	520	31.03	
Period of diagnosis							0.0520
1990–1999	627	32.97	88	38.94	539	32.16	
2000–2009	838	44.06	98	43.36	740	44.15	
2010–2013	437	22.98	40	17.70	397	23.69	
Site							< 0.001
Tongue	1218	64.04	198	87.61	1020	60.83	
Other parts of mouth	635	33.39	26	11.50	609	36.34	
Lip	49	2.58	2	0.88	47	2.8	
Smoking history							0.0352
Smoker	767	40.33	102	45.13	665	39.68	
Never	807	42.43	78	34.51	729	43.50	
Unknown	328	17.25	46	20.35	282	16.83	
Alcohol use history							0.056
Drinker	1120	58.89	142	62.83	978	58.36	
Never	407	21.40	27	11.95	380	22.67	
Unknown	375	19.72	57	25.22	318	18.97	
T classification							0.3065
T1	548	28.81	74	32.74	474	28.28	
T2	731	38.43	77	34.07	654	39.02	
T3	198	10.41	19	8.41	179	10.68	
T4	411	21.61	55	24.34	356	21.24	
Unknown	14		1	0.44	13	0.78	
N classification							0.6639
N0	1249	65.67	150	66.37	1099	65.57	
N1–3	547	28.76	61	26.99	486	29	
Unknown	106	5.57	15	6.64	91	5.43	
Distant metastases invasion							0.8439
M0	1895	99.63	225	99.56	1670	99.64	
M1	7	0.37	1	0.44	6	0.36	
Tumor stage extension							0.6774
Stage I–II	964	50.68	117	51.77	847	50.54	
Stage III–IV	823	43.27	93	41.15	730	43.56	
Unknown	115	6.05	16	7.08	99	5.91	
Treatment							
Surgery only	1028	54.05	94	41.59	934	55.73	< 0.001
Surgery with RT/CT	547	28.76	96	42.48	451	26.91	< 0.001
RT/CT	327	17.19	36	15.93	291	17.36	0.1153

Bivariate analysis of the independent variables was done using the Chi-square test to compare characteristics between the two groups. *P* value < 0.01 indicates a statistically significant difference. *CT* chemotherapy, *RT* radiotherapy

## Results

A total of 1902 patients comprised the study cohort. Those who were older than 85 years, younger than 18 years, or died within 6 months of diagnosis were excluded. The clinicopathologic characteristics of the two groups are presented in Table 1. Among these patients, 226 (11.88%) were less than 40 years of age (young group). Overall, the average patient age was 54.6 ± 12.2 years and 33.5 ± 4.7 years in the young group and 57.5 ± 9.9 years in the older group. In the young group, the morbidity rate of females was significantly higher than that in the older group (40.27% vs. 31.03%). The tongue was the most common primary site (64.04%), followed by other parts of the mouth. The tongue SCC in young patients appeared to be more common than in the older group (87.61% vs. 60.83%, *p* < 0.001). No statistical differences were found between the two groups with regard to tobacco and/or alcohol use. There was no significant difference between the groups concerning TNM classification or tumor stage. Interestingly, compared with the older group, the young group was less likely to undergo surgery alone (94, 41.59% vs. 934, 55.73%, *p* < 0.001). Young patients tended to receive postoperative adjuvant radiotherapy (RT) and/or chemotherapy (CT) compared with the older group (96, 42.48% vs. 451, 26.91%, *p* < 0.001).

**Fig. 1:**
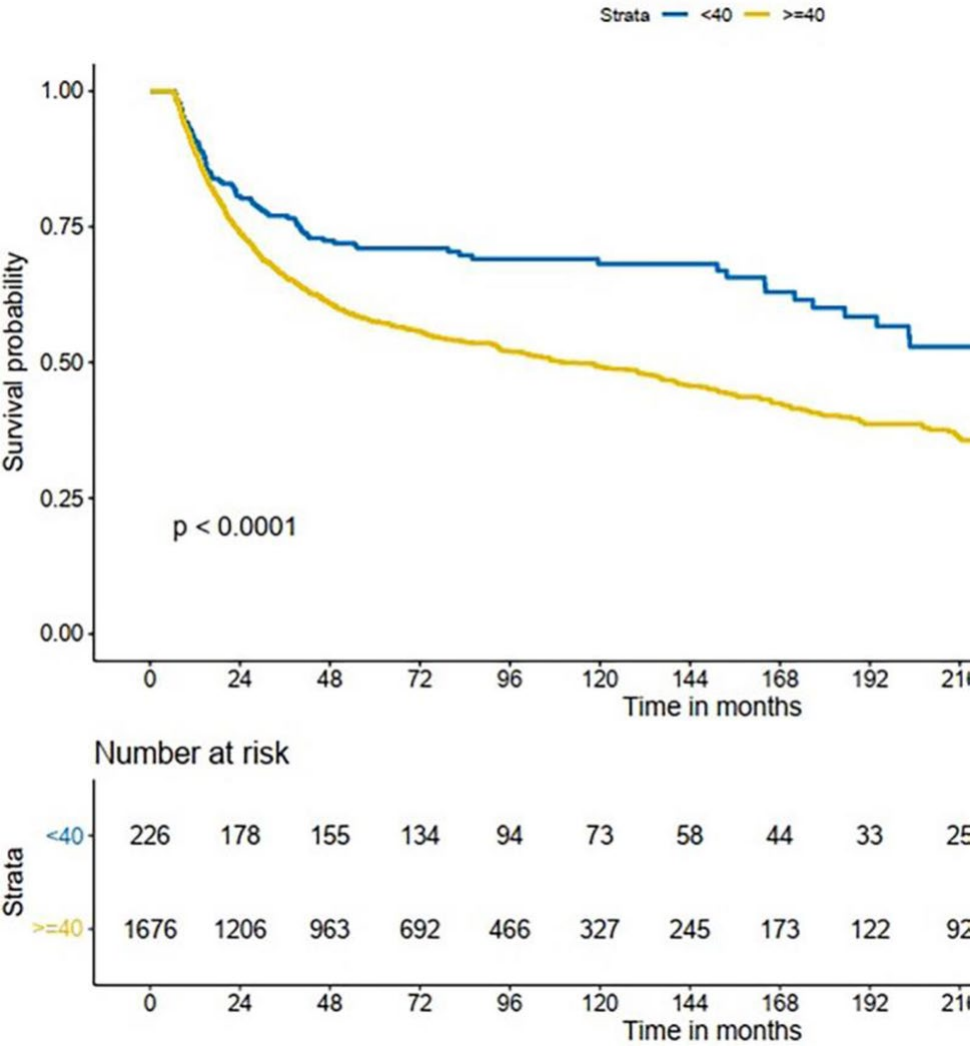
Kaplan–Meier estimate of overall survival between the age groups

**Fig. 2:**
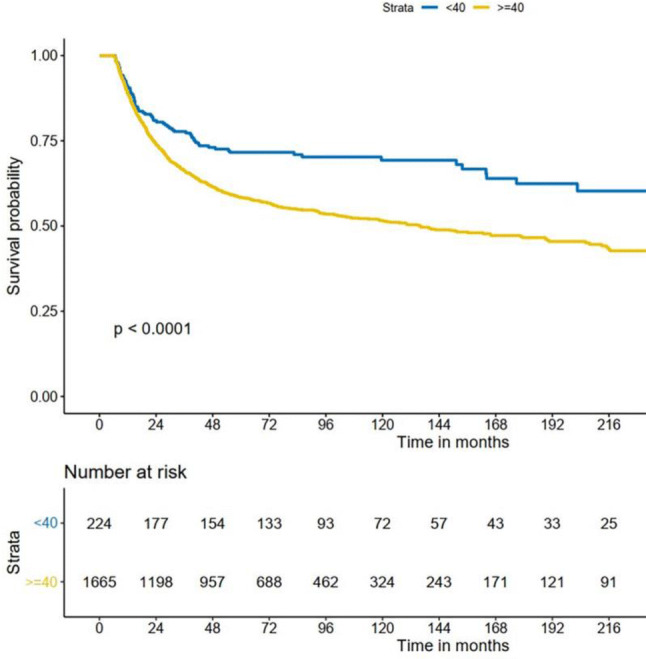
Kaplan–Meier estimate of disease-specific survival between the age groups

The minimum duration of follow-up time was 5 years. Of 1902 patients, the 5-year overall survival (OS) rate was 59% [95% confidence interval (CI) 56.8–61.2%] and the 5-year disease-specific survival (DSS) rate was 60% (95% CI 57.8–62.2%). The 5-year OS rate was 71% (95% CI 68.0–77.0%) in the young group and 57% (95% CI 54.6–59.4%) in the older group. The 5-year DSS rate was 72% (95% CI 67.0–78.0%) in the young group and 58% (95% CI 55.6–60.4%) in the older group. The OS and DSS between the two groups were compared with Kaplan–Meier plot. The results indicated that there were significant differences between the two groups. Figures 1 and 2 show that both OS and DSS in younger patients with OC-SCC are better than in older patients.

The univariate and multivariable Cox proportional hazards model for DSS were performed (Table 2). Older age was a significant predictor of worse prognosis at all stages. In addition, other clinically significant worse predictors of DSS in both univariate and multivariable regression included male sex, tumor site (other part of mouth), higher TNM classification, worse tumor stage, and treatment with RT/CT.

**Table 2:** Univariate and multivariate analyses of clinicopathologic and treatment factors for DSS

	Univariate		Multivariate	
	HR (95% CI)	*p*	HR (95% CI)	*p*
Age				
Young group (ref)	1		1	
Older group	1.685 (1.321–2.149)	< 0.001	1.593 (1.240–2.047)	< 0.001
Sex				
Male(ref)	1		1	
Female	0.695 (0.596–0.809)	< 0.001	0.910 (0.758–1.093)	0.3130
Period				
1990–1999 (ref)	1		1	
2000–2009	0.856 (0.737–0.994)	0.0420	0.852 (0.710–1.023)	0.0857
2010–2013	0.667 (0.546–0.813)	< 0.001	0.694 (0.56–0.861)	< 0.001
Site				
Tongue (ref)	1		1	
Other site of OC	1.855 (1.618–2.126)	< 0.001	1.389 (1.202–1.604)	< 0.001
Lip	0.447 (0.239–0.836)	0.0117	0.470 (0.249–0.888)	0.0201
Smoking history				
Smoker (ref)	1		1	
Never	1.349 (1.166–1.561)	< 0.0001	0.992 (0.817–1.203)	0.9311
Unknown	0.898 ( 0.726–1.111)	0.3211	0.951 (0.620–1.458)	0.8172
Alcohol use history				
Drinker (ref)	1		1	
Never	1.365 (1.164–1.602)	0.0001	1.097 (0.906–1.328)	0.3443
Unknown	0.869 (0.719–1.050)	0.1457	0.870 (0.587–1.290)	0.4899
T classification				
T1(ref)	1		1	
T2	1.800 (1.482–2.187)	< 0.001	1.454 (1.191–1.775)	< 0.001
T3	2.840 (2.225–3.625)	< 0.001	1.540 (1.133–2.094)	< 0.001
T4	3.590 (2.942–4.380)	< 0.001	1.727 (1.313–2.271)	< 0.001
Unknown	1.488 (0.658–3.365)	0.3399	1.065 (0.253–4.489)	0.9315
N classification				
N0 (ref)	1		1	
N1-3	2.480 (2.149–2.861)	< 0.001	1.684 (1.350–2.102)	< 0.001
Unknown	2.548 (1.988–3.265)	< 0.001	0.985 (0.180–5.391)	0.9865
Distant metastases				
M0 (ref)	1		1	
M1	5.704 (2.706–12.021)	< 0.0001	3.118 (1.453–6.688)	0.0035
Tumor stage				
Stage I–II (ref)	1		1	
Stage III–IV	2.613 (2.258–3.023)	< 0.001	2.425 (2.111–2.785)	< 0.001
Unknown	2.827 (2.198–3.634)	< 0.001	1.061 (0.187–6.017)	0.9466
Treatment				
Surgery only (ref)	1		1	
Surgery with RT/CT	1.698 (1.443–1.999)	< 0.001	1.297 (1.088–1.546)	< 0.001
RT/CT	4.606 (3.904–5.435)	< 0.001	3.156 (2.605–3.824)	< 0.001

A Cox proportional hazards model was used to analyze predictors of survival. *P* value < 0.01 indicates a statistically significant difference

**Fig. 3:**
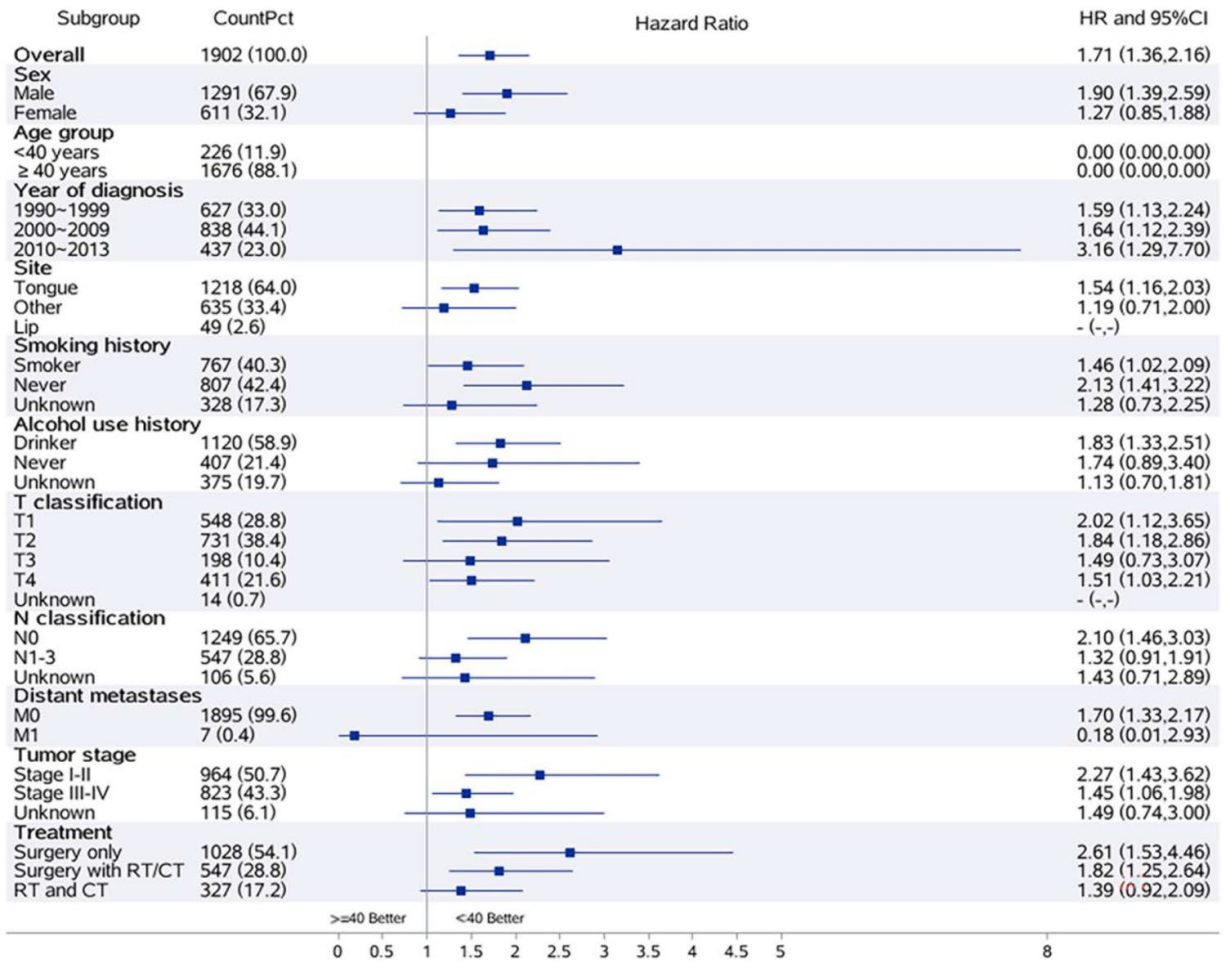
Subgroup analyses

The DSS in the young group was consistently favorable [all hazard ratios (HR) except distant metastases M1] across all subgroups (Fig. 3).

## Discussion

In many types of cancer, age at diagnosis is viewed as an independent predictor of outcome [11]. In the field of OC-SCC, there is no uniform category of “young” patients and previous analyses were performed using age thresholds ranging from 30 to 45 years of age [12–19]. It is difficult to determine a reasonable cutoff between “young” and “old” adults. Because 40 years of age was used as an age threshold in most of the previous studies, it is reasonable that for our study, a young adult is defined as someone less than 40 years of age. The inconsistent cutoff age for young patients has contributed to the conflicting findings in the literature. Therefore, there is a need for studies to use a standard division.

A review of the reported studies demonstrates that our clinical characteristic findings are in agreement with several previously published large cohort studies [12–19]. First, OC-SCC mainly occurs in men between the 5th and 6th decades of life. Second, the young group exhibited a higher proportion of women compared with adults 40 years and older (40.27% vs. 31.03%, respectively). The findings should heighten the awareness of the occurrence of OC-SCC in young women and merits further investigation. Third, lymph node (28.76%) and distant (0.37%) metastases are not unusual and a majority of patients had early stage disease (Stage I–II, 50.68%). Furthermore, more than half of patients (54.05%) were treated with surgery alone, as surgical treatment is the main treatment method. In the young group, patients were more likely to receive adjuvant RT and/or CT (42.48% vs. 26.91%, *p* < 0.01). In the findings on site predilection was generally consistent with the previous OC-SCC literature [3, 22, 23]. The young group was more likely to have tongue cancer (87.61% vs. 60.83%, *p* < 0.01).

Several similar studies have analyzed survival of patients with early-onset OC-SCC (Table 3). More population-based studies with large samples are needed, especially performed in non-western regions to estimate the outcome of OC-SCC in young groups of patients. In this study, it is notable that 11.88% of patients were diagnosed before age of 40 years, a higher rate than that in most studies, particularly those studies from the US and Europe [20, 24–26]. Considering that cancers of the oral cavity are highly frequent in southern Asia, the higher proportion in young people might indicate that the sociocultural lifestyle of the population, such as betel quid chewing and the use of tobacco and alcohol, plays an important role in this geographic or regional diversity [27]. In our study, the percentage of patients with early stage (Stage I–II, 50.68%) was lower than in western developed countries [20], but higher than other southern Asian regions (e.g., India, Thailand, Taiwan, and Japan) [11, 14, 17, 19, 28–30]. It is suggested that low socioeconomic status or related patient factors (e.g., education, diet, health care, and living conditions) may increase the risk of OC-SCC. A significant difference in survival rates was found among young people between affluent and non-affluent groups [31]. A deeper knowledge of the public on OC-SCC could help avoid exposing people to the risk factors. This means we need to do more to raise awareness by identifying people at risk and taking measures to allow early detection and minimize the undesirable consequences of OC-SCC [32].

**Table 3:** Similar studies that used a cutoff age of 40

Author	Year	Patients	Young group	Male:Female in young	Survival		Prognosis for young group
					Young	Old	
Udeabor et al. [26]	2012	977	3.9%	3.8:1	66.2%/5-y OS	57.6%/5-y OS	Better
Van Monsjou et al. [25]	2013	1762	3.1%	1.8:1	58%/5-y OS 69%/5-y DSS	42%/5-y OS 74%/5-y DSS	No significant difference in DSS; better for OS
Fang et al. [43]	2014	176	8.5%	0.7:1	63%5-y DSS	625-y DSS	No significant difference
Sun Q [15]	2015	486	7.2%	1.6:1	65%5-y DSS	66.75-y DSS	No significant difference
Jae-Ho Jeon et al. [37]	2016	117	20%	1.9:1	40%/5-y OS 42%/5-y DSS	70%/5-y OS 73%/5-y DSS	Worse
Mahmood et al. [11]	2018	115	34.8%	4.7:1	62.5%/5-y OS	37.3%/5-y OS	Better
Oliver et al. [20]	2019	22 930	9.9%	1.15:1	79.6%/5-y OS	69.5%/5-y OS	Better

Historically, numerous previous reports on this topic found that the biologic behavior of OC-SCC in younger patients was more aggressive compared with that in elderly patients [13, 34]. It has been reported that OC-SCC in young patients had a significantly higher rate of nodal metastases, which resulted in a more advanced tumor stage [20, 34, 35]. In clinicopathologic features, some studies have shown OC-SCC in young patients had a more advanced TNM classification and higher proportion of poorly differentiated tumors [36, 37]. However, reviewing the most recent studies, the treatment outcomes of the young group are heterogeneous and it is possible to confirm that younger patients may have similar or better outcomes than older patients [18, 20, 26, 38–44]. In our cohort, histopathologic variables, such as tumor TNM, did not show any significant differences between young and old patients. And, it was not possible to confirm a higher rate of nodal metastases. Therefore, it was no surprise that no difference was found in tumor stage between the two groups (*p* = 0.677). Interestingly, treatment comparisons showed that the younger patients were more likely to receive postoperative adjuvant RT and/or CT, a more aggressive approach than older patients. RT, generally recommended as an adjuvant treatment in head and neck cancers, is preferred for those with evidence of adverse features or advanced stage. For the patients involved in our study, CT alone is not recommended as a postoperative adjuvant therapy without RT. However, in rare cases, personal reasons, for example, misconceptions in irradiation, economic problem and rejection of so many times of radiotherapy fractions, could explain that they underwent CT alone as an adjuvant therapy without RT. It is unclear why young patients undergo intensification. A possible reason is that the young patients were more tolerant to adjuvant therapy than the old group and practitioners probably think young patients could benefit from treatment intensification despite there being no clear indication for adjuvant therapy.

The 5-year OS was 59% and the 5-year DSS rate was 60%. The 5-year OS was 71% in the young group and 57% in the older group. The 5-year DSS was 72% in the young group and 58% in the older group. The higher OS and DSS in young patients showed a significant prognostic advantage in younger patients. In both univariate and multivariable analyses, older age, advanced TNM stage, surgery with RT and/or CT, and RT and/or CT were associated with worse prognosis. In DSS subgroup analyses, the results of the better survival of the young group, as compared with the older group, were that treatment outcome was consistently favorable across all patient subgroups (except distant metastases M1). In clinical practice, age, stage, and site are the most important determinants of treatment selection for patients with OC-SCC [45]. However, these outcome data of the present study suggest that young age alone should not alter treatment.

There are limitations to our study. First, it was conducted at a single institution. Another limitation is lack of matched controls and recurrence or clinicopathologic data (e.g., tumor grade, extracapsular extension, margins, and number of examined lymph nodes). However, despite these limitations, our study is one of the largest studies from a high-risk region of OC-SCC, including the high quality of the data, which enabled us to control for multiple factors and minimize error and bias.

## Conclusion

OC-SCC in young adults represents a rare disease, with an increasing incidence, particularly in females. Comparison by age group showed no differences in rates of advanced TNM stage. But young adults are more commonly treated with adjuvant RT and/or CT. Our findings have evidenced that young age at diagnosis is an independent predictor of better survival. Thus, a comprehensive tailoring of treatment on a case-by-case basis according to current guidelines is recommended.
